# Evaluation of the accuracy, safety, utility and feasibility of using an urgency self-assessment application in self-referred patients in the emergency department: study protocol for a prospective, multicenter cohort trial

**DOI:** 10.1186/s13063-025-09101-4

**Published:** 2025-10-31

**Authors:** Daniela Krüger, David Legg, Dorian Jouhoff, Larissa Eienbröker, Nora Köhne, Konrad Neumann, Martin Möckel, Anna Slagman

**Affiliations:** 1 Department of Emergency and Acute Medicine, Campus Virchow-Klinikum and Campus Charité Mitte, Charité - Universitätsmedizin Berlin, Berlin, Germany; 2 Institute of Biometry and Clinical Epidemiology, Campus Charité Mitte, Charité - Universitätsmedizin Berlin, Berlin, Germany

**Keywords:** SmED, Self-referred patients, Low-acuity consultation, Emergency department, Symptom assessment application (SAA), Mobile application, Patient navigation, Utility, Accuracy, Safety, Feasibility

## Abstract

**Background:**

Algorithm-based patient navigation is a key feature of the emergency and acute care reform being discussed in Germany. The software Structured Initial Medical Evaluation in Germany (SmED) is designed to assist in determining the appropriate time for medical complaints to be treated, as well as their most appropriate level of care. SmED is available in three different configurations, each of which is currently used in the German acute care sector and can be utilized by either a provider (SmED-Contact, SmED-Contact +) or a self-applicant (SmED-Patient). SmED-Patient is offered as a web-based self-assessment application that provides recommendations on the medical urgency and appropriate level of care for acute symptoms. This is the first study to explore and evaluate the accuracy, safety, utility and feasibility of using the self-assessment application SmED-Patient for self-referring patients and medical staff in the emergency department (ED) setting in Germany.

**Methods:**

The study uses a mixed methods approach, including a prospective, multicenter cohort study combined with retrospective expert review of SmED-Patient recommendations for all cases by an expert panel as well as focus groups and a microsimulation. Expert reviews assess SmED-Patient recommendations on patients’ treatment urgency and the appropriate level of care based on routine clinical data. Adult patients (≥ 18 years) who self-refer at two inner-city emergency departments in Berlin (Germany) and able to provide written informed consent will be invited to participate. Target number of patients is *n* = 150. The primary endpoint is the accuracy of SmED-Patient’s recommended level of care, measured as the agreement with the expert review for all cases. Secondary endpoints include safety, utility and feasibility of use. Data sources include primary data, routine clinical data, and qualitative data from focus groups and a microsimulation.

**Discussion:**

This study will provide insight into the accuracy, utility, safety and feasibility of using the self-assessment application SmED-Patient in the ED. By facilitating medical self-assessment for self-referring walk-in patients, SmED-Patient could contribute to re-directing patients to ambulatory care providers, improving the efficiency of ED operations and benefit providers’ as well as patients’ care experiences in the ED.

**Trial registration:**

German Clinical Trials Register: DRKS00036266. 25/02/2025.

**Supplementary Information:**

The online version contains supplementary material available at 10.1186/s13063-025-09101-4.

## Administrative information

**Table:** 

Title {1}	Evaluation of the accuracy, safety, utility and feasibility of using an urgency self-assessment application in self-referred patients in the emergency department: study protocol for a prospective, multicenter cohort trial
Trial registration {2a and 2b}	German Clinical Trials Register
Protocol version {3}	2025–04-30 Version 1.1
Funding {4}	The study is financed by the Central Institute for the Statutory Health Insurance System (Zi) in Germany
Author details {5a}	Daniela Krüger (1), David Legg (1), Dorian Jouhoff (1), Larissa Eienbröker (1), Nora Köhne (1), Konrad Neumann (2), Martin Möckel (1), Anna Slagman (1) (1) Department of Emergency and Acute Medicine, Campus Charité Mitte & Campus Virchow-Klinikum, Charité Universitätsmedizin – Berlin (2) Institute of Biometry and Clinical Epidemiology, Campus Charité Mitte, Charité Universitätsmedizin – Berlin
Name and contact information for the trial sponsor {5b}	Prof. Dr. rer. medic. Anna Slagman Charité - Universitätsmedizin Berlin Charitéplatz 1 10117 Berlin Tel.: + 49 30 450 565 659 E-Mail: anna.slagman@charite.de
Role of sponsor {5c}	The study is being led and initiated by the sponsor.

## Introduction

### Background and rationale {6a}

In contrast to a gatekeeper system, patients in non-gatekeeper systems, such as Germany, are under much greater pressure to make appropriate healthcare choices [see 1]. As a result, patients may not always present to the most appropriate provider. In Germany, around 45% of emergency department (ED) patients present without referral based on self-assessment of symptoms [2]. A recent study of routine clinical data collected in German EDs demonstrated that 33.2% of all analyzed ED presentations were retrospectively classified as “low acute” [3]. This suggests that some of these cases may not have required emergency care and could have been effectively managed at an alternative level of care. Even though these cases are unlikely to cause ED crowding, this patient group is especially affected by long waiting times in a crowded ED situation which negatively impacts patient and provider satisfaction and results in a higher number of patients left without being seen [4]. In addition, low acuity patients may not find the optimal care for their needs in EDs. In the German case, ED physicians can neither issue sick notes, nor prescribe reimbursable medication for Statutory Health Insurance (SHI) patients, and as in general, EDs do not provide long-term care (see [5]). ED interaction is also characterized by a particular lack of time, likely putting pressure on patients to communicate symptoms and receive advice on health issues [6]. In light of these considerations, the establishment of standards and tools for safe and effective patient navigation at appropriate levels of care is a desirable policy goal, and its use is increasingly supported by healthcare providers and policy actors [7–11]. However, there is currently no single endorsed algorithm-assisted decision support system for patient navigation in Germany, and evidence base on their accuracy, safety, utility and feasibility of use is still limited [12, 13].

The leading example of widespread pilot use of an algorithm-based patient navigation support system in Germany is the Structured Initial Medical Evaluation in Germany (SmED). SmED is a German configuration based on the validated assessment system SMASS (Swiss Medical Assessment System) [14]. Its configuration SmED-Contact is used by providers for telephone-based assessments as part of the on-call medical service of the Regional Association of SHI Physicians (available in Germany on 116117). The SmED-Contact + configuration is being piloted by providers with direct patient contact, such as ambulance services, outpatient practices and hospitals with integrated acute outpatient care [13]. Providers and policymakers alike expect the primary benefits of a generalized use of algorithm-based patient navigation to be efficient and safe patient allocation, contributing to improved cost-effectiveness and reducing the resource demands associated with emergency and acute care settings [12].

The current utilization, research, and debate surrounding algorithm-assisted patient navigation support systems primarily focus on the use by healthcare professionals. While the evidence base for these systems is growing [15], it is even more scarce for algorithm-assisted patient self-assessment and navigation in acute and emergency care settings. This finding is unexpected in light of the growing popularity of Symptom Assessment Applications (SAAs) among laypeople [16, 17] and their purported advantages for self-assessment and self-navigation, particularly in complex, non-gatekeeper systems.

Early studies suggest that the use of SAAs by laypersons to self-assess the urgency of symptoms is beneficial and produces more accurate results than unassisted self-assessments [18]. Further studies have shown that using a SAA as a triage tool in EDs can enhance safety [14], potentially improving operational efficiency. At the same time, further research has highlighted inconsistencies in urgency level assessments between healthcare providers and SAAs [19]. Notably, most existing studies focus primarily on patient safety, often relying on patient vignettes rather than real-world data [20].

While the emphasis on patient safety is crucial, it tends to overshadow other potential benefits of implementing SAAs in the ED. For instance, SAAs could support ED consultations by preparing patients for symptom-centered conversations, which may ultimately facilitate patient-provider interactions [21]; SAAs could also serve to bridge patients’ lack of health and system literacy, facilitating future decisions about healthcare utilization. In order to address these and the above research gaps and to integrate them into an overall research agenda, the present study examines the accuracy, safety, utility and feasibility of utilizing an algorithm-based symptom self-assessment application for self-presenting patients and their healthcare providers in the ED, conducting a prospective, multicentered, mixed-methods cohort trial. It uses SmED-Patient as another SmED configuration currently applied by the on-call medical service of the SHI Physician’s Association in Germany (available at https://www.116117.de/de/patienten-navi.php). This web-based tool for self-assessing the urgency of symptoms is comparable to other tools offered by international acute and emergency care providers (e.g., 111 online by the NHS, https://111.nhs.uk/). It is part of the SmED “system family,” using the same algorithm but is designed for lay use in the preclinical setting.

This is the first study to thoroughly evaluate the accuracy, safety, utility and feasibility of using SmED-Patient in the ED setting for both ED patients and providers in Germany.

### Objectives {7}

#### Primary objective

The primary objective of the study is to measure the accuracy of the SmED-Patient recommendation for the patient care level by analyzing its agreement with the recommendation of the expert panel for all cases (see Table 3).

#### Secondary objectives

1. To investigate SmED-Patient’s recommendation and symptom assessment compared to the retrospective symptom assessment of attending ED doctors and the utility of the SmED-Patient symptom short report in the ED for a sub-sample of *n* = 30–60 cases (*symptom assessment overlap and utility*).
2. To investigate the agreement of recommendations between SmED-Patient and SmED Contact + (*SmED rating agreement*).
3. To investigate the proportion of cases in which the SmED-Patient recommendation is assessed to be potentially patient endangering or inappropriate by experts (*safety and appropriateness*).
4. To investigate SmED-Patient’s utility, including its comprehensibility, usability, response confidence, response time, satisfaction, trust and hypothetical compliance as well as preparedness for the medical consultation by patients, including subgroup analysis to explore aspects that influence patients’ assessment, e.g., health literacy, age (*patient utility*).
5. To investigate the utility of SmED-Patient to providers in the ED setting, including practical utility of use and perceived benefits of a SmED-Patient implementation in the ED (*provider utility*).
6. To investigate the proportion of disagreement between the patient self-assessment of urgency without decision-support and the SmED-Patient assessment of urgency (*patient urgency*).
7. To investigate the feasibility of implementing SmED-Patient in the ED setting (*feasibility*).

### Trial design {8}

The study is a prospective, multicentre cohort study of adult self-referred patients in the ED to assess the accuracy of the SmED-Patient recommendation as compared to an expert panel (gold standard). A parallel mixed-methods approach is chosen to compare, integrate and validate the research results through triangulation for inclusion of different data sources, data methods and conceptual perspectives [22].

## Methods: participants, interventions and outcomes

### Study setting {9}

The study is a multicenter, ED-based trial with two study sites in Berlin, Germany. The study sites are the EDs of a large tertiary care hospital, representing an ED with standard care and an ED that integrated standard ED care with outpatient care (“fast track”) provided by an ED physician.

### Eligibility criteria {10}

The inclusion and exclusion criteria for the prospective, multicenter cohort study were selected in a manner that was as inclusive as possible in order to avoid any potential for selection bias. The criteria focus on patients who are potential users of a tool like SmED-Patient, reflecting the conditions under which such a tool might be used in the acute and emergency care context (e.g., low-acuity complaints). Inclusion and exclusion criteria for participation in the prospective cohort study are as follows:

- Inclusion criteria
  1. Age ≥ 18 years
  2. Self-referred walk-in presentation at one of the participating EDs
  3. Ability to perform the SmED-Patient assessment and the prospective survey (adequate German or English language skills, verbal communication possible, adequate cognition, etc.)
- Exclusion criteria
  1. Minor age
  2. Medical referral or medical transport to the ED
  3. Insufficient knowledge of the German or English language to understand the information about the study contents, provide consent and participate in the study
  4. Insufficient cognitive status to understand the information about the study contents and to consent to the study
  5. Legal guardianship (due to potential conflicts with time-sensitive study measures if the legal guardian is not present)
  6. Patients requiring immediate medical care (e.g., unstable, intubated patients)
  7. Prior medical contact with a physician in the ED

The inclusion and exclusion criteria were assessed through prescreening in in the ED’s digital documentation system (E.CARE ED) and through verbal interactions with patients by trained study personnel.

### Who will take informed consent? {26a}

Eligible patients will be asked to provide verbal and written informed consent by the study staff following ED triage. A copy of the signed informed consent form, approved by the Research Ethics Committee of the Charité – Universitätsmedizin Berlin, will be provided to the participant, as well as an information leaflet about the study, including information about data protection, participants’ rights and relevant contact information. Participants may withdraw from the study at any time without affecting their treatment. The informed consent process is conducted in accordance with the Declaration of Helsinki and complies with the EU General Data Protection Regulation (GDPR).

For patients who decline to participate in the study their reasons for not participating will be anonymously documented and results will be reported descriptively. This allows for insights into the selection bias of the study and the characterization of patient groups unsuitable for performing self-assessments of symptoms in the ED setting using SmED-Patient.

### Additional consent provisions for collection and use of participant data and biological specimens {26b}

Participants in the study measures of module 2 to 4, consisting of an expert panel, focus group interviews, a microsimulation and their evaluation (see Fig. 2) will give verbal and written informed consent. Participants will be offered a copy of the signed consent form and an information leaflet about the study measure. Both the informed consent forms and the information leaflets for each study measure have been approved by the Research Ethics Committee of the Charité—Universitätsmedizin Berlin.

Nurses will be asked to provide verbal informed consent for the pilot study of nurse assessment of urgency in the ED.

No biological samples will be taken that go beyond standard care.

## Interventions

### Explanation for the choice of comparators {6b}

This is an observational study that does not involve a clinical intervention. During the study, there will be no treatment intervention based on the recommendation of SmED-Patient or SmED-Contact +. ED treatment will follow usual routine care. As part of the study measures, the study provides a comparison and assessment of the overlap in results between two SmED configurations (SmED-Patient, SmED-Contact +) as well as an expert panel recommendation regarding appropriate care level.

### Intervention description {11a}

The software “Strukturierte medizinische Ersteinschätzung in Deutschland” (SmED) is designed to assist in determining the appropriate time to treat (see Table 1) as well as the most appropriate point of care (see Table 2). Tables 1 and 2 provide an overview of the urgency and care levels defined by the SmED manufacturer (manufacturer: in4medicine AG, Bern, Switzerland; Importer: Health Care Quality Systems [HCQS], Göttingen, Germany).

**Table 1:** Level of urgency according to “time to treat” and corresponding treatment recommendations

Time to treat	Name	Recommended action
1	Emergency	CPR readiness. There is a potentially life-threatening condition. Medical treatment must be given now.
2	Immediately	Medical treatment does not allow any delay. Treatment should be given immediately.
3	Today	Medical treatment does not have to take place immediately. Medical treatment should take place within the next 24 h.
4	Later	Medical treatment is not urgent. If the symptoms do not subside in the next 2 days, treatment by a physician is indicated.

Legend: *CPR* Cardiopulmonary resuscitation

**Table 2:** Level of care according to “point of care” and corresponding treatment recommendations

Point of care	Name	Recommended action
1	EMS	Medical treatment should be provided by the Emergency Medical Service (EMS).
2	Hospital/ED	Medical treatment should be provided at a hospital in an ED.
3	Physician	Medical treatment should be provided by a registered outpatient care physician.
4	Tele-consultation	The person concerned should be advised in a teleconsultation on how to proceed.

Legend: *ED* Emergency department, *EMS* Emergency medical service

Participants in the multicentre cohort study will be assessed by study personnel using SmED-Contact + and asked to self-assess their symptoms using SmED-Patient. The order of the assessments is randomized. The assessments are conducted using a tablet, with the assessments being processed and stored in a web-based study entity provided by the Central Institute for Statutory Health Insurance in the Federal Republic of Germany (Zi).

An expert panel (blinded to the SmED patient's recommendation) will recommend the level of care for each case, before the expert panel is presented with the SmED patient’s recommendations for the level of care to assess the appropriateness and risk of the recommendation for the patient’s safety (see Table 7). The comparison between SmED-Patient and SmED-Contact +, the latter conducted by a provider, provides additional validation of patient self-assessment with SmED-Patient.

SmED is a Class IIB medical product certified according to the Medical Device Directive (MDD). The study is conducted in accordance with the Declaration of Helsinki and relevant principles of ICH-GCP. ISO 14155 has been considered where applicable (informed consent, data protection, certified training for SmED-Contact + users), though full compliance is not required as SmED is used within its intended use.

### Criteria for discontinuing or modifying allocated interventions {11b}

Study personnel will assess participants’ ability to complete the SmED-Patient assessment and prospective survey prior to study enrollment. If participants have moderate difficulty completing the study measures, they may receive assistance from study personnel (e.g., technical support). In the event of severe difficulty in performing and completing the relevant study measures, study staff will consider the case exclusion from the study.

### Strategies to improve adherence to the interventions {11c}

Not applicable.

### Relevant concomitant are permitted or prohibited during the trial {11d}

Study participants are asked not to use any other symptom checkers during the study period of t0 in the ED.

### Provisions for post-trial care {30}

Not applicable.

### Outcomes {12}

#### Primary outcome

Accuracy, measured as percentage of the perfect agreement in the assessment of the binary level of care between SmED-Patient and the expert panel for patients who self-referred to the ED.

SmED-Patient gives recommendations to the level of care in the following categories (see also Table 2):

- Emergency Medical Service (EMS),
- Hospital Emergency Department (ED),
- Registered outpatient care physician,
- Teleconsultation.

For the purposes of the study, the categories are combined into two levels, as these reflect the sectoral separation and empirically existing levels of care in Germany:

- Emergency Care: ED, EMS
- Outpatient Care: Registered outpatient care physician, teleconsultation (performed by a registered outpatient care physician).

The primary endpoint will be operationalized as a comparison of the level of care recommended by SmED-Patient and the expert panel for each case (see Table 3). Given that directing patients to the most appropriate level of care is the central purpose of algorithms like SmED, the primary endpoint of this study focuses on the perfect agreement between the recommendations of SmED-Patient and the expert panel in terms of binary care level only. The recommended time to treat will be examined as a secondary endpoint, as it is primarily relevant for patients with complaints suitable for outpatient care [10].

**Table 3:** Operationalization of the perfect agreement assessment

		**Expert panel**	
		**Emergency care**	**Outpatient care**
**SmED-Patient**	**Emergency care**	**Perfect agreement**	Mismatch
	**Outpatient care**	Mismatch	**Perfect agreement**

Legend: Emergency care = ED, EMS; outpatient care = outpatient care physician, telemedicine

#### Primary null hypothesis

The perfect agreement on the binary care level between SmED-Patient and expert panel is less than or equal to 58.5%.

#### Secondary outcomes

The secondary outcomes are as follows:

- Symptom assessment overlap and utility: Assessment of recommendation and symptom assessment by SmED-Patient compared to a retrospective physician assessment and the utility of SmED-Patient in the ED (physician assessment in a sub-sample of *n* = 30–60 cases; based on an adapted version of a validated assessment sheet [10]).
- Agreement of SmED ratings: Percentage of agreement between the recommendations of SmED-Patient and SmED-Contact +.
- Patient safety and appropriateness: Percentage of cases in which the SmED-Patient recommendation is judged by the experts to be appropriate, appropriate but too cautious, or inappropriate and potentially hazardous (judged according to a range of appropriateness) (see Table 7).
- Patient assessment of urgency: Percentage of disagreements between patients’ self-assessed urgency and SmED-Patient assessed urgency (see Table 4).
- Patient utility: Evaluation of utility, including comprehensibility, usability, confidence with responses, response time, satisfaction, trust and hypothetical compliance as well as preparedness for the medical consultation. Subgroup analysis: Investigation of aspects that influence patients’ assessment of SmED-Patient, e.g. health literacy, age.
- Provider utility: Qualitative evaluation of the utility, including practical utility and perceived benefits of the SmED-Patient short report and of SmED-Patient (full assessment) by ED personnel in focus groups.
- Feasibility: Qualitative assessment of the feasibility of implementing SmED-Patient in the ED setting.

**Table 4:** Overview of target variables and corresponding survey instruments

Target variable	Instrument
Reasons for ED presentation	Measured using an instrument designed by the research group to identify reasons and motives (see Additional file 1)
Patients’ assessment of urgency	Measured using SmED’s categorization of urgency: 1) Emergency (Immediately); 2) As soon as possible; 3) Within 24 h; 4) More than 24 h [13]
Health literacy	HLS19-Q12 (sub-dimension for coping with illness, items 1 to 4) [23], HLS19-COM-P-Q6 (short version) [24]
Sources of information for symptom assessment prior to ED presentation	Measured using an adapted instrument tested within the research group [25]
Medical care received for presented symptom(s) prior to ED presentation	Measured using an adapted instrument tested within the research group [25]
Sociodemographic and socioeconomic baseline data	Validated instruments from established survey panels (e.g., SOEP & ALLBUS) [26, 27]
Patients’ perspective on utility of SmED-Patient in the ED	Measured using the G-MAUQ-S [28], supplemented by dimensions of comprehensibility, confidence with responses, trust and hypothetical compliance as well as preparedness for the medical consultation
Hypothetical compliance with SmED-Patient advice	Prompted with a patient vignette for influenza and advice to seek outpatient care within 24 h, based on [20]
Physicians’ perspective on utility of SmED-Patient in the ED	Measured using an adapted instrument tested within the research group [13]

Legend: *ALLBUS* German General Social Survey, *SOEP* Socio-Economic Panel Germany, *G-MAUQ* German version of the mHealth App Usability Questionnaire, *HLS19-Q12* Instrument to measure General Health Literacy, *HLS19-COM-P-Q6* Instrument to measure Communicative Health Literacy in interaction with physicians, *ED* emergency department

### Participant timeline {13}

**Fig. 1:**
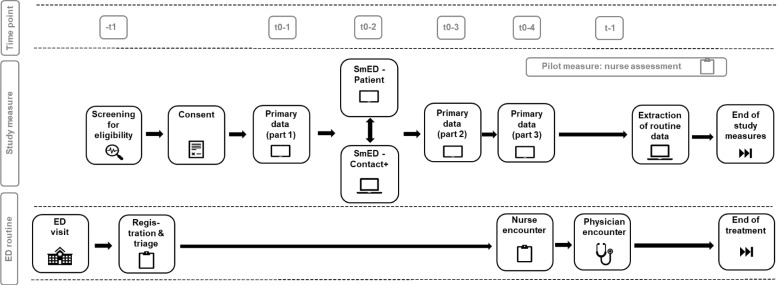
Study measures at baseline (t0)

The study period for participants in the multicenter cohort study extends from ED admission and study enrollment to the end of the study measures. Primary data collection at t0 is divided into three parts at four time points, including the collection of primary data prior to the use of SmED-Patient (t0-1) and after the use of SmED-Patient (t0-2 and t0-3). The timeline of study measures at baseline (t0) is illustrated in Fig. 1. T0-1 includes patients’ perceived urgency, which is sensitive to the bias of the SmED assessments, and precedes the randomized SmED assessments at t0-2; part 2 at t0-3 includes patients’ utility assessment of SmED-Patient; part 3 at t0-4 includes patients’ reasons for ED presentation and questions on health literacy, characterisation of patients’ complaints, and socio-demographics (see Table 8).

### Sample size {14}

We expect a perfect agreement on the binary care level between SmED-Patient and the expert panel in 70% of the cases. To demonstrate that this agreement exceeds 58.5%, we aim to apply a one-sided binomial test with over 80% power at a significance level of alpha = 0.025. The threshold of 58.5% reflects the minimum level of agreement considered acceptable. Based on this assumption, a sample size of 150 evaluable patients was calculated. Assuming a dropout rate of 25%, a total of up to 200 patients will be enrolled in the multicenter cohort study. The sample size planning was conducted using R version 4.2.1.

The estimate for a perfect agreement on the binary care level is based on the available literature, from which studies using symptom assessment applications and either gold standard vignettes or physician review of cases concur at 62%−63% [29, 30]; reviews and vignette studies found that the triage accuracy of SAAs ranged from 33 to 98%, with the majority of studies relying on vignettes rather than prospective data collection [18, 20, 30]. In this study, we assume 70% agreement and therefore that there will be a higher level of agreement between SmED-Patient and the expert panel than is suggested by the results of previous studies [29, 30]. This is because the assessment of agreement will be limited to the binary care level (i.e., ED or outpatient practice), rather than, for example, four or five triage categories. In case the agreement falls below the expected threshold, qualitative data from providers on the perceived utility of symptom self-assessment can be used to help contextualize the findings. In addition, quantitative data from both providers and patients such as urgency assessments and patient evaluations of SmED-Patient (e.g., ease of use) can support interpretation and provide further insight into potential discrepancies.

For the focus groups, staff from the two participating EDs will be invited to participate in two focus groups (*n* = 2 with each *n* = 5 participants). The focus groups will consist of staff from differently structured EDs to ensure a broad range of perspectives and implementation scenarios in German emergency care: Standard care in the ED, separate low-acuity fast track within the ED (“fast track”). No specific sampling is required for the microsimulation.

### Recruitment {15}

The recruitment follows approaches that have been used in similar studies [21]: Study nurses determine patients’ eligibility for the study through a pre-screening in the ED’s digital documentation system (E.CARE ED). Eligible patients will be approached in the ED waiting area after triage and before the first physician encounter. If eligible patients verbally express interest in participating in the study, study nurses will verbally review inclusion and exclusion criteria and initiate the consent process, including explanation and information about the study, answering questions, and obtaining written consent. Among all ED patients, adult German- and English-speaking patients will be included who present themselves at the ED on a walk-in and self-referral basis and who do not require immediate medical treatment. Patients are eligible if ED staff ask them to wait in the waiting area, indicating no need for immediate ED treatment. No formal triage system is used for the eligibility screening. This approach ensures that a wide range of complaints and urgency levels can be considered.

The prospective patient survey as well as the application of SmED-Patient and SmED-Contact + takes place during the waiting time for the first physician encounter on the day of presentation in the ED. To assist with patient recruitment, student assistants will be involved in the recruitment process. They will receive structured training in patient recruitment and the use of SmED-Contact +. The recruitment period is tentatively planned to be 6 months but will continue until the target number of patients (*n* = 150) is reached.

Recruitment for the focus group interviews will be conducted concurrently with recruitment for the cohort study by distributing flyers and informing ED staff about the study and the focus groups during team time-outs in the ED. Focus group participants will receive a 100 euros voucher for their participation, and an additional 50 euros for evaluating the microsimulation. The microsimulation will be performed by the study team, including students from the Charité-Universitätsmedizin Berlin. Students are recruited through the university’s internal mailing list and are offered a 50 euros voucher for their participation. Experts for the panel will be approached by the study team on the basis of their medical experience in working in the ED or ambulatory setting. Experts receive compensation, including 2,500 euros for written evaluations of 50% of cases and an additional 1,250 euros for case conferences in the event of disagreement. For each of these study measures, interested potential participants will be informed of the study verbally and in writing before written consent is obtained.

## Assignment of interventions: allocation

### Sequence generation {16a}

Patients who participate in the study will be assessed by SmED-Contact + and are asked to conduct a self-assessment using SmED-Patient. In order to control for the potential reciprocal impact on patient responses to the SmED-Patient and SmED-Contact + assessments, the sequence of the two SmED configurations is randomized using RStudio (Version 2024.09.1) and is secured. Block randomization is used for this purpose. The randomization protocol for the multicenter cohort study is implemented in REDCap (block randomization: *n* = 8 per block) and is known to the study coordinator and principal investigator.

The panel of experts will conduct retrospective case reviews of all valid cases.

### Concealment mechanism {16b}

The allocation is unknown to the study staff until the moment the patients confirm their written consent to the study and the study staff starts the digital recruitment documentation in REDCap.

### Implementation {16c}

The study staff initiates the allocation process in REDCap by pressing the randomization button as part of the randomization module in REDCap. The system allocates the patient based on a preloaded block randomization list (*n* = 8 per block), stratified by the two study centers and implemented within REDCap.

## Assignment of intervention: blinding

### Who will be blinded {17a}

The study personnel will be blinded to the SmED-Contact + results to reduce any potential cross-influence on the assessments. To this end, the study will use a version of SmED-Contact + that does not display the assessment recommendation. Patients are blinded to the results of the SmED-Patient recommendation to reduce uncertainty about the recommendation and not to influence patient behaviour. Instead, hypothetical compliance will be asked based on a patient vignette. Study personnel can access recommendation in retrospect through an assessment archive.

The results of the SmED-Contact + and SmED-Patient assessments will be retrieved from a web-based study entity provided by the Zi (see 18a).

### Procedure for unblinding if needed {17b}

Not applicable. Intervention is unblinded and sequence is known after randomization.

## Data collection and management

### Plans for assessment and collection of outcomes {18a}

**Fig. 2:**
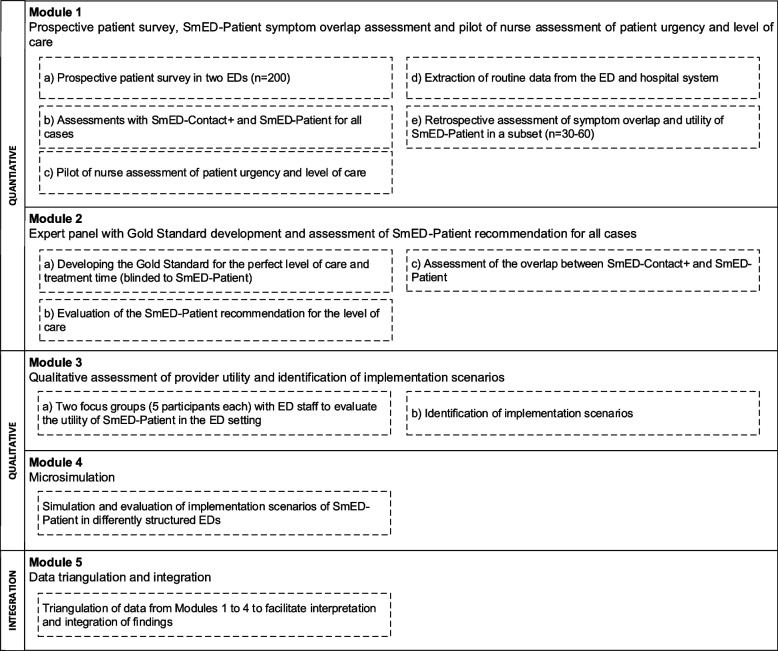
Module overview

The project consists of five working modules (see Fig. 2 for an overview).

#### Module 1: prospective patient survey, retrospective subgroup SmED-Patient symptom overlap and utility assessment, and pilot of nurse assessment of patient urgency and level of care

The first study measures, including the study enrollment, primary data collection, application of SmED-Patient and SmED-Contact +, will be performed in the waiting area of the ED while waiting for ED treatment (see Fig. 1). The primary data collection at baseline is divided into 3 parts, of which part 1 (t0-1) includes an instrument that is sensitive to the influence of the SmED assessments (patient self-assessment of urgency, see Table 4) and part 2 (t0-3) includes instruments that are sensitive to the physician encounter (e.g. reasons for ED presentation, see Table 4). Part 3 is not considered bias-sensitive. It can be collected independently of the time of the physician encounter.

The prospective cohort study will measure the following outcomes using the following instruments:

After the study enrollment and the first primary data collection (t0-1), the assessment with SmED-Patient and SmED-Contact + will take place preferably before the first contact with the treating physician. According to the objectives of the study, both applications are used to measure the agreement between both SmED versions. The assessment with SmED-Contact + is expected to take 2–3 min and will be performed by the study personnel. Assessments are conducted exclusively by study personnel with certified training in the use of SmED-Contact +. The application of SmED-Patient is done by the patients themselves and takes on average 3–4 min. The results of both SmED assessments will be blinded to the patient and the study personnel as well as clinical personnel. The order of the SmED applications will be randomized in order to control the possible mutual influence on the response behavior of the patients.

In a pilot study, the treating ED nurses are tasked with assessing the patient’s treatment urgency and the level of care required, based on the initial medical assessment (triage) (see Fig. 1). An adaption of a validated assessment sheet [13] is employed to assess the treatment urgency (according to Table 1) and the appropriate level of care (according to Table 2). Table 5 outlines the criteria utilized by the nurse as well as their evaluation basis in making this assessment.

**Table 5:** Attending nurse assessment of treatment urgency and care level

Assessment sheet	Evaluation basis
• Assessment of mobility	• Vital parameters, triage category, lead symptom, overall clinical impression and anamnesis
• Assessment of general condition	
• Assessment of treatment urgency and appropriate care level	

Furthermore, in a sub-group of *n* = 30–60 patients, ED physicians evaluate the utility of SmED-Patient retrospectively. They therefore receive a summary (short report) of the SmED-Patient assessment as well as all routine ED data as well as the further hospital course in case of admitted patients. The ED physicians provide an evaluation of the digital anamnesis and symptom assessment generated by SmED-Patient in terms of completeness and appropriateness of the questions asked. They also assess the utility of the SmED-Patient summary for assessing patients’ complaints and treatment urgency in routine ED care. For this purpose, a separate assessment sheet has been developed. This assessment is conducted subsequent to the patient’s treatment and is based on a comparison of the symptom summary provided by SmED-Patient and the medical routine data. Table 6 outlines the criteria utilized by the physicians in making this assessment, as well as the variables derived from the routine data set.

**Table 6:** Attending physician evaluation of the symptom assessment and the utility of the SmED-Patient summary

Assessment sheet	Evaluation basis
• Evaluation of completeness, appropriateness and utility of SmED-Patient symptom assessment summary	• Vital parameters
	• Triage category
	• Leading symptom and medical anamnesis
	• ED diagnostics, diagnoses, treatment as well as data from inpatient stays and discharge data from the ED and hospital
	• SmED-Patient symptom assessment (summary version)

Legend: *ED* emergency department, *SmED* Structured Initial Medical Evaluation Assessment Germany (German: Strukturierte Medizinische Ersteinschätzung Deutschland), *SmED-Patient* Self-Assessment Version of SmED

Finally, routine data from the ED and hospital documentation system are extracted.

The order of the study measures is as follows: (1) primary data collection part 1 (t0-1), (2) use of the SmED assessments in randomized order: SmED-Patient and SmED-Contact +, preferably prior to the first encounter with the treating physician (t0-2); (3) primary data collection part 2 (t0-3) following the SmED-Patient assessment and potentially after first contact to the treating physician; (4) primary data collection part 3 (t0-4), and (5) assessment by the treating nurse at a variable time during the ED stay; (6) extraction of routine data by the study personnel at the end of the clinical case. The physician’s assessment (7) of the patient sub-sample *n* = 30–60 is conducted retrospectively after the completion of clinical data extraction (see Fig. 2).

Mandatory study measures for a case to be considered valid include the SmED-Patient assessment and regular termination of ED treatment and extracted routine data.

#### Module 2: expert panel assessment of SmED-Patient recommendation for all cases

An independent expert panel, consisting of primary care physicians (n=2) and physicians working in the ED (n=2), evaluates all valid cases in pairs.The cases will expectedly be divided equally between the pairs, with each case being evaluated independently by two experts. First experts are blinded to the SmED-Patient recommendations and only provided with the initial clinical patient data (routine ED and hospital data). Based on these data the experts provide (1) their own rating of perfect care level and treatment time along the evaluation matrix and (2) their rating of a range of appropriateness of the combination of care levels and treatment times along the evaluation matrix (Table 7). Thereafter the experts are unblinded to SmED-Patient recommendations and evaluate (3) the appropriateness of the SmED-Patient recommendation and (4) potential hazards to patient safety by the SmED-Patient recommendations, both in comparison to the perfect care level and time based on their former rating and the range of appropriate combinations of level of care and treatment time.

**Table 7:** Range of appropriateness for SmED-Patient recommendations assessed by an expert panel

			**Expert panel**			
			**Emergency care**	**Outpatient care**		
			*Emergency (immediate or as soon as possible)*	*As soon as possible*	*Within 24 h*	> *24 h*
**SmED-Patient**	**Emergency care**	*Emergency (immediate or as soon as possible)*	Appropriate	Appropriate but too cautious	Appropriate but too cautious	Appropriate but too cautious
	**Outpatient care**	*As soon as possible*	Inappropriate and potentially hazardous	Appropriate	Appropriate but too cautious	Appropriate but too cautious
		*Within 24 h*	Inappropriate and potentially hazardous	Inappropriate and potentially hazardous	Appropriate	Appropriate but too cautious
		> *24 h*	Inappropriate and potentially hazardous	Inappropriate and potentially hazardous	Inappropriate and potentially hazardous	Appropriate

Legend: Care levels = bold and underlined; treatment time = italics

The expert panel’s assessment is based on the SmED-Patient recommendation, the patient’s complete medical history from the ED, and the clinical data from the hospital documented both in the study database using REDCap and through pseudonymized copies of clinical documents (e.g., lab results, physician’s letter). The expert panel assessments are performed using REDCap. After the physician experts have reviewed their share of cases, an interim analysis is performed to detect any discrepancies in [1] rating regarding care level and treatment time, and [4] rating regarding hazards to patient safety between the two experts. Criteria for inclusion in the case conference are: a) a discrepancy between one expert and the other in identifying the perfect level of care and treatment time as resulting from the combination of “emergency care + emergency”, “outpatient care + as soon as possible”, “outpatient care + within 24 h” or “outpatient care + > 24 h”; and b) a discrepancy in the categorization of a case as “inappropriate and potentially hazardous” by one expert and “appropriate” or “appropriate but too cautious” by the other (Table 7). In the event of these discrepancies, a case conference is held with the participation of another expert, who acts as a moderator, to seek agreement on the discrepant ratings.

It should be noted that experts are independent physicians who will be remunerated for their contributions.

#### Module 3: qualitative assessment of provider utility and implementation scenarios

The aim of the focus groups is to obtain a qualitative assessment of the utility and applicability from a healthcare provider perspective for two different models of emergency care. It aims to deepen the understanding of the healthcare provider perspective through a dynamic exchange of perspectives and needs between different healthcare professions regarding a hypothetical implementation of SmED-Patient in two different emergency care models.

In addition, multiple context-sensitive implementation scenarios will be identified and documented. Participants will also be asked to relate ideas for potential improvement in the application of SmED-Patient in the ED. Two focus groups (*n* = 5 each) are planned for this study. Focus groups will be guided by a semi-structured interview guideline, allowing participants to discuss their experiences, challenges, and perceptions of SmED-Patient and its potential implementation in the ED setting. The focus group interview guideline will be developed through an iterative process, drawing on the literature from implementation studies [31, 32], as well as the experience of the research group. Focus groups are led and moderated by a member of the research team.

Focus groups will be recorded and transcribed verbatim, with data analysed using qualitative content analysis [33]. Results will be reported according to the standards for reporting qualitative research guideline [34].

#### Module 4: microsimulation study and qualitative assessment of implementation scenarios (feasibility)

The identified implementation scenarios for SmED-Patient in the ED from the focus groups (module 3) will be converted into a simulation script by the study team. The microsimulation takes place as a filmed enactment by and with study personnel and students in the study group’s facilities and has no impact on routine ED operations. Enacted patients will not be familiarized with the use of SmED-Patient prior to the microsimulation. The evaluation of the filmed microsimulation will be carried out in a final workshop where the microsimulation will be screened and all participants of the previous focus groups (*n* = 5–10) will be invited to discuss the following questions and provide context for their assessments:

1. Does the filmed microsimulation match the suggested implementation scenario by the focus groups (scenario translation)?
2. Does the use of SmED-Patient in the ED setting appear realistic regarding ED workflow and patient-provider interactions (realism)?
3. Does the use of SmED-Patient in the ED setting appear feasible regarding workflow integration (feasibility)?
4. Does the use of SmED-Patient in the ED setting appear beneficial for ED workflows and patient-provider consultations (benefit)?

The goal of the microsimulation is the quasi-realistic simulation of the implementation of SmED-Patient in the ED and its qualitative evaluation by the participants of the focus groups (module 3). The final workshop will be audio-recorded and supplemented with notes from the research team members guiding the workshop and observing participants’ reactions to the filmed microsimulation. The analysis follows the approach described in module 3.

Table 8 provides an overview of the schedule of events and assessments in modules 1–4 of the study.

**Table 8:** Schedule of enrolment, interventions and assessments

Timepoint	Study period											
	**Module 1 (patient survey)**									**Module 2 (expert panel)**	**Module 3 (focus group interviews)**	**Module 4 (micro-simulation)**
	**Screening**	**Baseline**				**Treatment**		**Follow-up**				
	*** − t1***	***t0-1***	***t0-2***	***t0-3***	***t0-4***	***t1 (nurse enconter)***	***t2 (physician encounter)***	***t3 (post-discharge)***	***t4 (retro-spectively)***	***t5***	***t6***	***t7***
**Eligibility**												
Eligibility screening	X											
Informed consent	X									X	X	X
**Interventions**												
Primary data (part 1)		X										
Randomization of order of SmED assessments + SmED assessments			X									
Primary data (part 2)				X								
Primary data (part 3)					X							
Nurse assessment						X						
Physician assessment									X			
Expert panel										X		
Focus group interviews											X	
Microsimulation												X
Usual care						X	X					
**Assessments**												
Baseline variables		X										
Demographics	X	X										
SmED assessments			X									
Nurse assessment						X						
Primary outcome			X							X		
Secondary outcomes		X	X	X	X				X		X	X
Routine clinical data								X				

*t* Timepoints of study measure, *SmED* Structured Initial Medical Evaluation Assessment Germany (German: Strukturierte Medizinische Ersteinschätzung Deutschland)

#### Module 5: data triangulation and integration

The objective of the module is to triangulate the data from modules 1 to 4, with a view to advancing the interpretation and integration of the findings as well strengthening their validity from different stakeholder perspectives. In particular, the evaluation of the benefits to patients with different social characteristics and providers of different professional backgrounds of using SmED-Patient within the context of the ED will be analysed. This will allow us to draw inferences about the overall benefits of using the system in acute and emergency care. In order to achieve this, a mixed-methods matrix will be constructed [35], combining the results of the qualitative and quantitative data collection on the respective endpoints (i.e. patient and provider utility) to ascertain the overall benefit and the emerging themes.

### Plans to promote participant retention and complete follow-up {18b}

The study period for each participant ends after t0. No follow-up is required.

### Data management {19}

The study group will be provided with a web-based research application by the Zi. This application contains a demo version of both SmED-Patient and SmED-Contact +. This ensures an independent test of the applications without interfering with the live results, which the Zi collects online from users of the SmED-Patient configuration “Patient Navi” (https://www.116117.de/de/patienten-navi.php). Assessment results and assessment IDs can be retrieved from the SmED research application and linked to primary and secondary data at a later stage. The primary and secondary data will be documented in an electronic case report forms (eCRF) using the REDCap database, which is hosted on the internal servers of the Charité—Universitätsmedizin Berlin. Upon reasonable request, aggregate data will be provided.

The study complies with the General Data Protection Regulation (GDPR). All data will be pseudonymized to allow linkage with the assessment results from SmED-Patient and SmED-Contact + as well as the expert panel reviews. All data will be stored on a secure, restricted university server with access limited to study personnel. The study’s data management plan has been reviewed and approved by the Clinical Trials Office, an independent body of Charité – Universitätsmedizin Berlin.

### Confidentiality {27}

Access to the trial data at participating study sites will be limited to the principal investigator and authorized study team members. Recruitment logs will be securely stored in locked cabinets at the local sites. Following the guidelines of the German Research Foundation, data will be archived for a period of 10 years [36].

### Plans for collection, laboratory evaluation and storage of biological specimens for genetic or molecular analysis in this trial/future use {33}

Not applicable.

## Statistical methods

### Statistical methods for primary and secondary outcomes {20a}

Two-sided 95% confidence intervals are calculated for the proportion of perfect agreement on the binary care level (primary endpoint). The primary null hypothesis is rejected if the lower bound of the confidence interval is greater than 58.5%. In addition to the primary endpoint measure, the kappa measure is calculated to assess the agreement between SmED-Patient and the expert panel.

The statistical analysis will be multifocal in accordance with the stated endpoints. All data will be analysed using the Statistical Package for Social Sciences (IBM SPSS V 30.0.0.0 [171]). Descriptive analysis includes the presentation of relative and absolute frequencies for categorical variables and the calculation of median and interquartile range (non-normal) or mean and standard deviation (normal). The agreement of SmED ratings between SmED-Contact + and SmED-Patient is performed using the Intraclass Correlation Coefficient (ICC) similar to Cohen’s Kappa to measure the level of agreement between the two sets of recommendations.

### Interim analyses {21b}

An interim analysis is performed in Module 2 after both physician experts have reviewed all cases to identify any discrepant assessments of the primary endpoint (perfect level of care) and the secondary endpoint patient safety, that require a consensus process with a third expert in a case conference.

### Methods for additional analyses (e.g. subgroup analyses) {20b}

Exploratory subgroup analyses and logistic regression (assuming model-specific conditions are met) are used to test relationships between relevant sociodemographic and socioeconomic data and study endpoints. Study cohort characteristics in terms of demographical data will be compared between the two study sites to assess potential differences using descriptive statistics analysis (relative and absolute frequencies; median and interquartile range or mean and standard deviation, depending on the distribution).

### Methods in analyses to handle protocol non-adherence and any statistical methods to handle missing data {20c}

Protocol non-adherence (e.g., presence of a doctoral referral) in the multicenter cohort study will result in case exclusion.

### Plans to give access to the full protocol, participant-level data and statistical code {31c}

Anonymous data as well as statistical code will be made available upon reasonable request.

## Oversight and monitoring

### Composition of the coordinating center and trial steering committee {5d}

The coordinating center is the Charité – Universitätsmedizin Berlin. The study and its scientific evaluation is initiated and led by AS and performed by DJ, LE, NK and DK from the Health Service Research in Emergency and Acute Medicine at the Departments of Emergency Medicine Campus Charité Mitte and Campus Virchow-Klinikum Charité – Universitätsmedizin Berlin. Planning for sample size has been conducted by KN from the Institute of Biometry and Clinical Epidemiology of the Charité – Universitätsmedizin Berlin. In addition, Charité—Universitätsmedizin Berlin evaluates and monitors compliance with the data protection plan and the ethics plan.

### Composition of the data monitoring committee, its role and reporting structure {21a}

Not applicable. Data monitoring will be carried out by the study team (AS, DK).

### Adverse event reporting and harms {22}

Adverse events may be reported by participants via a free text field located at the conclusion of the questionnaire or directly to the study personnel, who will be responsible for documenting this information. Moreover, MM serves as the designated point of contact for the receipt of unexpected adverse event reports from patients and/or treating physicians. However, because the use of SmED-Patient and the other study interventions are considered to be low-risk, we do not expect any adverse events directly related to the study measures.

### Frequency and plans for auditing trial conduct {23}

The study will be monitored by AS and DK, including site visits during recruitment, plausibility checks of data and included cases for validity. An interim review of data and study documents will be performed after recruitment of *n* = 30 cases. A full review of data and study documents will be performed at the end of enrolment for the study.

### Plans for communication important protocol amendments to relevant parties (e.g. trial participants, ethical committees) {25}

Any protocol amendments must receive approval from the Ethics Committee of Charité – Universitätsmedizin Berlin. These changes will be communicated to the funding body and the protocol will be updated in the clinical trial registry accordingly. All study personnel will be informed of the changes, and study materials will be revised to reflect the updates.

### Dissemination plans {31a}

Dissemination through academic and public channels will be used to disseminate the results of this study. The results will be published in peer-reviewed journals, with preference given to open access journals to ensure broad accessibility. In addition, in order to engage with the scientific and practitioner communities, the results will be presented at national and international conferences.

## Discussion

To our knowledge, this is the first study to systematically evaluate patients’ self-assessment of symptoms using the Symptom-Assessment Application (SAA) SmED-Patient, while also examining the perceived utility of the application’s output by emergency care providers. The study includes two different ED settings to provide results that approximate real-world acute and emergency care in Germany. However, the study does not evaluate the utility and safety of using SmED-Patient in the preclinical setting. Nevertheless, the results of the study will provide insights into the benefits and feasibility of using SmED-Patient in the ED setting and into patient groups for whom the use of such an algorithm-based symptom assessment could be a safe and a beneficial support tool used during the waiting period. Results will be disseminated through peer-reviewed journal articles as well as national and international conferences. This may inform policy makers on the utility and safety of implementing such tools in routine acute and emergency care. By facilitating medical self-assessment for self-referred walk-in patients, SmED-Patient could help improve the efficiency of ED operations and the patient and provider care experience in the ED as well as support future healthcare decision-making of patients.

## Trial status

Recruitment is planned for a period of 6 months starting in March 2025. If necessary, recruitment will continue until the planned sample size of *n* = 150 is reached. Continuous data monitoring will be used to oversee recruitment progress and ensure data completeness. The total research time is set to 26 months (01.02.2024–31.03.2026).

## Supplementary Information

Additional file 1: Tables a and b.

## Data Availability

Anonymous data will be made available upon reasonable request.

## Abbreviations

ED: Emergency department
Zi: Central Institute for Statutory Health Insurance in Germany (German: Zentralinstitut für die kassenärztliche Versorgung in der Bundesrepublik Deutschland)
SHI: Statutory Health Insurance
SmED: Structured Initial Medical Evaluation Assessment Germany (German: Strukturierte Medizinische Ersteinschätzung Deutschland)
CPR: Cardiopulmonary resuscitation
SOEP: Socio-Economic Panel Germany
ALLBUS: German General Social Survey
G-MAUQ: German version of the mHealth App Usability Questionnaire
HLS19-COM-P-Q6: Instrument to measure Communicative Health Literacy in interaction with physicians
HLS19-Q12: Instrument to measure General Health Literacy
EMS: Emergency Medical Service
