# Supplementary material for: Evaluation of the accuracy, safety, utility and feasibility of using an urgency self-assessment application in self-referred patients in the emergency department: study protocol for a prospective, multicenter cohort trial
Source: Trials. 2025 Oct 31;26:463. doi: 10.1186/s13063-025-09101-4 (PMC12579394; doi:10.1186/s13063-025-09101-4)
Supplement: Supplementary file 1 — Additional file 1: Tables a and b. [file 13063_2025_9101_MOESM1_ESM.docx]

Additional file 1

Table a: Presentation of the target variable “Reasons for ED presentation” (see Table 4; developed by the research team)

| *Introduction*  We begin with the questions about your reasons for visiting the emergency department today.  People come to the emergency department for different reasons. The following questions relate to your reasons for visiting the emergency department today. On a scale from “fully applies” to “does not apply at all”, to what extent do the following statements apply to the reasons you came to the emergency department today? | | |
| --- | --- | --- |
| No. | *Items* | *Response* |
| 1 | I think my symptoms are serious. | Fully applies  Somewhat applies  Rather does not apply  Does not apply at all  *Do not know*  *No answer* |
| 2 | I think I need medical care that I can only get in the emergency department at the moment. |  |
| 3 | Medical care in a GP or specialist practice was difficult to obtain (e.g. no appointment). |  |
| 4 | I was unable to visit a practice for personal reasons (e.g. state of health, opening hours). |  |
| 5 | I think that the medical care here in the emergency department is better than in a GP or specialist practice. |  |
| 6 | I came to the emergency department on the recommendation or referral of other people or institutions.  *If Item 6 “fully applies” or “somewhat applies” – participants are asked to answer item 6_1 (see table b)* |  |
| 7 | It was easier for me to come here to the emergency department. |  |
| 8 | If you have received treatment:  I am dissatisfied with the treatment of my complaints to date.  (Otherwise select “no answer”) |  |
| 9 | If you have a practice: I do not have enough trust in my general practitioner or specialist.  (Otherwise select "no answer") |  |
| 10 | Other reason: |  |

Table b: Presentation of Item 6_1

| No. | *Items* | *Response* |
| --- | --- | --- |
| 6_1 | Which of the following people or institutions recommended that you visit the emergency department today?  *Multiple answers possible* | *Checkboxes* |
| 6_1_1 | Recommendation from family members/friends |  |
| 6_1_2 | Recommendation from school/work |  |
| 6_1_3 | Recommendation from other hospital departments/outpatient clinics |  |
| 6_1_4 | Recommendation from another medical professional (e.g., nurse, pharmacist) |  |
| 6_1_5 | Recommendation from a doctor in an outpatient practice |  |
| 6_1_6 | Medical recommendation for follow-up examination in the emergency department |  |
| 6_1_7 | *Do not know* |  |
| 6_1_8 | *No answer* |  |
